# The Impact of School Closures on Learning and Mental Health of Children: Lessons From the COVID-19 Pandemic

**DOI:** 10.1177/17456916231181108

**Published:** 2023-07-10

**Authors:** Deni Mazrekaj, Kristof De Witte

**Affiliations:** 1 Department of Sociology, Utrecht University; 2 Nuffield College, University of Oxford; 3 Faculty of Economics and Business, KU Leuven; 4 United Nations University – Maastricht Economic and Social Research Institute on Innovation and Technology (UNU-MERIT), Maastricht University

**Keywords:** school closure, COVID-19 pandemic, children, learning, mental health

## Abstract

To curb the spread of the coronavirus, almost all countries implemented nationwide school closures. Suddenly, students experienced a serious disruption to their school and social lives. In this article, we argue that psychological research offers crucial insights for guiding policy about school closures during crises. To this end, we review the existing literature on the impact of school closures during the COVID-19 pandemic on children’s learning and mental health. We find that the unprecedented scale and length of school closures resulted in a substantial deficit in children’s learning and a deterioration in children’s mental health. We then provide policy recommendations on how to ensure children’s learning and psychosocial development in the future. Specifically, we recommend that more attention should be paid to students from marginalized groups who are most in need of intervention, evidence-informed and personality-tailored mental-health and social- and emotional-learning programs should be implemented in schools, and generational labels should be avoided.

The COVID-19 pandemic has disrupted children’s education at an unparalleled global scale and speed. In an effort to mitigate the spread of the coronavirus, schools were suspended in more than 190 countries, affecting more than 1.6 billion learners (UNESCO, 2021a). In 2020, schools were closed for 79 instruction days on average, which is equivalent to almost half a school year in many countries. The extent of school closures differed by country, with schools being fully closed for 53 instruction days on average in high-income countries and 115 days in lower-middle-income countries. In the United States, nearly all of the 55 million children in kindergarten through 12th grade were affected by school closures (Golberstein et al., 2020).

Although the impact of COVID-19 on education has been severe, in a way never seen before in previous epidemics, emergencies, or crises (UNESCO, 2021b), school closures are not a new phenomenon. They have occurred in many countries as a result of adverse weather events and teacher strikes, among others, and a substantial education and economics literature indicates that school closures generally lead to large attainment deficits. For instance, Marcotte and Hemelt (2008) found that 5 days of school closures caused by snow in the U.S. state of Maryland led to a 3% lower reading and math achievement than in years with no school closures. Likewise, teacher strikes in the Canadian province of Ontario led to a reduction of 29% of a standard deviation in Grades 3 to 6 mathematics test scores (Baker, 2013). School closures may also have important long-run effects. Exploiting variation in the prevalence of teacher strikes in Argentina, Jaume and Willén (2019) found that children who were exposed to teacher strikes in primary school were more likely to drop out of high school, had 2% to 3% lower earnings, and had a higher probability of unemployment. In addition, high school dropout is believed to instigate various undesirable social outcomes, such as crime, poor health, lower life expectancy, and lower overall happiness (Oreopoulos & Salvanes, 2011).

Given the large negative impact school closures are likely to have on children, some authors have highly debated the usefulness of school closures during the COVID-19 pandemic. For instance, Townsend (2020) argued that school closures may incite loneliness and mental-health issues, dramatically increasing suicide risk among children and adolescence and leading to more deaths than would have occurred because of the coronavirus. Townsend (2020) stated,

Such bigger picture thinking has been curiously absent in policy-making throughout this crisis, which has infuriated many academics who understand and work with risk. . . . This is a global disaster in the making, and frankly, we should be collectively ashamed of our neglect of young people during this time. Our children are the very people who will bear the brunt of the economic fall out of the pandemic lockdown and are also the ones being most damaged by it. (p. 265)

Likewise, the significantly altered lives of children on a global scale have led many media outlets to name the young generations affected by COVID-19 as “Generation C” (Yong, 2020) or even as “The Lost Generation” (Hill, 2020), suggesting that these generations are “scarred for life.”

Clearly, there are limits to the analogy between school closures caused by teacher strikes and adverse weather events on the one hand and school closures caused by the COVID-19 pandemic on the other. Nonetheless, the initial COVID-19 responses have been developed with little guidance from psychological research. In this article, we argue that psychological research offers crucial insights for guiding policy about school closures during crises. For this purpose, we first discuss the available evidence on the impact of the COVID-19 school closures on children’s learning. As an inclusion criterion, we consider systematic reviews and meta-analyses of the empirical studies conducted since the outbreak of the pandemic. Then, we consider the literature on the impact of the COVID-19 school closures on children’s mental health using the same inclusion criterion. Finally, we provide recommendations that may guide policymakers to ensure children’s learning and psychosocial development in the future.

## School Closures and Children’s Learning

### Learning deficit

School closures during the COVID-19 pandemic may have negatively affected children’s learning through either a delay in expected learning progress or a loss of already obtained knowledge. In line with the literature (Engzell et al., 2021; Gambi & De Witte, 2021; Maldonado & De Witte, 2022), we encompass both by using the term “learning deficit.” To date, several systematic reviews have been conducted that investigated the impact of COVID-19 school closures on children’s learning (for a comprehensive review of the effect of the pandemic on education as a whole, see also Daumiller at al., in press). Hammerstein et al. (2021) reviewed 11 observational studies (five cross-sectional and six longitudinal) between March 2020 and April 2021 in four European countries, China, Australia, and the United States (only one of which was judged to have a low risk of bias) and found that school closures reduced children’s mathematics and reading achievement by about .10 *SD*. Likewise, De Witte and François (2023) reviewed 24 observational studies (three cross-sectional and 21 longitudinal) between 2020 and 2022 in 15 European countries (no risk assessment was conducted) and observed an average learning deficit of .11 *SD*.

Other studies found even larger learning deficits. Di Pietro (2023) conducted a meta-analysis of 38 observational studies (26 cross-sectional and 12 longitudinal) and one experimental study between 2020 and 2022 in 12 European countries, Australia, Brazil, China, Egypt, Mexico, South Africa, and the United States (six of which were rated to have a low risk of bias by the authors) and found that the learning deficit was .19 *SD*. This learning deficit is comparable with a learning deficit caused by Hurricane Katrina in the United States. Finally, a review of 42 observational studies (16 cross-sectional and 26 longitudinal) from March 2020 until May 2022 in nine European countries, Brazil, Colombia, Mexico, South Africa, and the United States (nine of which were rated to have a low risk of bias) showed an even larger overall learning deficit of about .14 *SD*, suggesting that students lost about 35% of a school year’s worth of learning (Betthäuser et al., 2023). Although the empirical evidence remains limited and often contradictory across countries, we observe in general that these learning deficits occurred for students in primary and secondary education alike. Longer school closures seem to have been associated with larger learning deficits, and learning deficits were highest in countries that have experienced a more severe course of the pandemic in terms of high incidence of infection and mortality. Students experienced learning deficits already in the first half of 2020, and these deficits remained constant throughout the pandemic.

Despite considerable investments in remedial practices such as summer schools, additional school tutoring, and investments in information and communication technology (De Witte & Smet, 2021), the findings indicate that the current policies have been unable to reverse the learning deficits that occurred early in the pandemic, at least until now. This is particularly concerning given that earlier studies have linked learning deficits early on to a higher risk of high school dropout (De Witte et al., 2013), which in turn has severe consequences in terms of earnings, health, and overall well-being (Oreopoulos & Salvanes, 2011). In fact, the U.S. Department of Education has estimated that the average high school dropout costs the economy approximately $272,000 over the person’s lifetime (McFarland et al., 2020). Likewise, delaying students with learning deficits for 1 or more years is very costly because grade retention leads to an annual earnings loss at age 28 of about €3,000 (8.5%) because of reduced labor-market experience (ter Meulen, 2023). Moreover, the heterogeneity among students makes implementing a similar practice more intricate. It is often also argued that schools should be lenient in track advice or grading. However, this might lower the motivation and engagement of students and lower the signaling value of a degree.

### Increased inequality

The systematic reviews above indicate that the pandemic exacerbated educational inequalities. Said otherwise, children from low socioeconomic backgrounds and low-income countries experienced the largest learning deficits. This is likely a result of the disparity in the access to technology to allow for continued remote education. Nearly one-third of adults in the United States with an annual household income of less than $30,000 do not own a smartphone, and more than 40% do not have broadband service or a home computer. African American children are 8% less likely to have high-speed Internet and 4% more likely to have no Internet access at all (Almeida et al., 2022). Even when having access to the Internet, children from low socioeconomic backgrounds often do not have a quiet place to study (OECD, 2017). Another reason for an increase in educational inequalities is that parents with a disadvantaged background may have been less able to help their children with their schoolwork (Maldonado et al., 2022). Parents with a disadvantaged background may have limited educational knowledge themselves and are therefore less able to support their children’s homeschooling. Even if they have an adequate educational background, parents with a disadvantaged background may not be able to take time away from income-generating activities to help their children (Carmichael et al., 2022). Parents with a disadvantaged background are also less likely to provide private tutoring to their children to compensate for learning deficits (Buchmann et al., 2010). Finally, additional factors may play a role in exacerbating inequalities in learning deficits as a result of school closures in developing countries. For instance, Ethiopian girls may be at higher risk of earlier marriage when they are not at school (McDougal et al., 2018).

## School Closures and Mental Health

### Loneliness, anxiety, and depression

Children generally rely on schools for social activities and strengthening competencies, and in many countries, such as the United States, schools offer their only hot meal for the day (Almeida et al., 2022). Closing schools induces substantial strain on both children and the parents to accommodate for the loss of all the school functions. For instance, the lack of socialization within schools is difficult to replace during general lockdowns, leading to an increase in loneliness. Loneliness has been found to be detrimental for mental health, leading to higher anxiety and depression up to 9 years later. In a systematic review of 41 observational studies (30 cross-sectional and 11 longitudinal) from January 2020 to June 2022 in six European countries, the United States, Australia, China, Hong Kong, Brazil, Israel, and Chile (six of which were rated 8 or 9 on a 9-point quality scale), Farrell et al. (2023) reported that higher rates of loneliness during the COVID-19 pandemic were associated with higher depression symptoms among children and adolescents.

In line with the research on loneliness, Viner et al. (2022) reviewed 36 observational studies (22 cross-sectional and 14 longitudinal) from January to September 2020 in four European countries, the United States, Canada, Brazil, China, Japan, India, and Bangladesh (13 of which were rated as high-quality by the authors) and found that the rates of anxiety and depression increased during the school closures, with little difference by socioeconomic status. For instance, a population-based study from the United Kingdom found that 53.3% of girls and 44% of boys ages 13 to 18 years had symptoms of anxiety and trauma above population threshold (Levita et al., 2020). Although loneliness is a significant risk factor for suicidal behavior (McClelland et al., 2020), school closures did not appear to have led to more suicides by youths, as evidenced by two high-quality, longitudinal, pre–post studies in England and Japan reviewed by Viner et al.

School closures have been associated with a range of adverse outcomes that may have contributed to mental-health issues. In most studies reviewed by Viner et al. (2022), children and adolescents have experienced a fall in physical activity and a rise in sedentary behavior. This has in turn been associated with worse mental health in young people (Biddle & Asare, 2011). Studies also suggest an increase in social media use that was in turn associated with anxiety and depressive symptoms (Y. Lee et al., 2022).

### The role of personality traits

The systematic review by De Witte and François (2023) also revealed that children’s experiences and behaviors during school closures may depend on personality. In Belgium, Iterbeke and De Witte (2022) found that more open children considered the period of school closures as an opportunity to learn new skills during the COVID-19 pandemic and that conscientious students embraced the remote learning. By contrast, children high in neuroticism experienced higher distress and felt more impaired during the crisis. More recently, the relationship between personality traits and coping with school closures has been confirmed in other countries as well (Iterbeke et al., 2022). As an underlying mechanism, personality traits, such as conscientiousness, have been associated with self-regulation learning strategies (Bidjerano & Dai, 2007) and problem-solving (Connor-Smith & Flachsbart, 2007), whereas extraversion is relevant for effective online collaboration (Borg et al., 2021).

### Parental stress

School closures can also have an indirect effect on children through their effect on the parents. The family-systems theory views parents and children as two interlinked systems (Kerr & Bowen, 1988). Events that affect parents will in turn affect their children and vice versa. How parents coped with the COVID-19 pandemic and how they coped with school closures will inevitably affect the psychosocial development of their children. In a review of 10 observational studies (seven cross-sectional and three longitudinal) from March 2020 until August 2020 in six European countries, the United States, China, and Japan (seven of which were rated “good” and none of which were rated “excellent” by the authors), Lehmann et al. (2021) found that parental stress surged, with parents reporting increased anxiety and depression. This is unsurprising given the added childcare responsibilities parents faced as a result of school closures while working from home and the already large worries related to financial difficulties and fears of getting sick or dying. In turn, this parental stress may have translated to a worse mental health of children. Indeed, in a meta-analysis of 18 observational studies (13 cross-sectional and five longitudinal) during the COVID-19 pandemic in five European countries and the United States (none of which were rated to have a low risk of bias by the authors, however), Stracke et al. (2023) found that increased parenting stress is associated with worse children’s mental-health outcomes.

### Child safety

Finally, increased parental stress may also lead to an increase in intimate-partner violence that may in turn translate to an increased risk of child maltreatment or violence toward children (J. Lee, 2020). For many students, school closures meant that children spent a greater proportion of their day with abusive parents. In a review of 32 observational studies (30 cross-sectional and two longitudinal) from the first wave of the COVID-19 pandemic in nine European, three South American, three North American, five Asian-Pacific, and two African countries (11 of which were rated as high quality by the authors), Kourti et al. (2023) found that domestic-violence cases surged during the COVID-19 pandemic in virtually all parts of the world. This is in line with the United Nations Secretary-General António Guterres’s call for measures during the pandemic to address a “horrifying global surge in domestic violence” directed toward women and girls (United Nations, 2020). Yet the rate of police and social services’s reports of child abuse has substantially declined during the COVID-19 pandemic. Much of this decline is likely due to school closures because schools are an important way in which child abuse can be identified. Indeed, Garstang et al. (2020) found that the proportion of child-protection medical referrals originating from schools approximately halved during the pandemic. Thus, school closures may have rendered child abuse to remain hidden during the COVID-19 pandemic.

## Discussion

The evidence on the impact of school closures during the COVID-19 pandemic on children’s outcomes suggests that school closures led to a deterioration in children’s mental health and a deficit in children’s learning. This is particularly worrisome because the school closures’ benefit for reducing mortality may be limited (Bayham & Fenichel, 2020). However, we note an important limitation of the reviews above. Specifically, none of the studies—regardless of the investigation of school outcomes or mental health—could address the potential confounding of the effect of school closures by the effect of the overall COVID-19 pandemic. It is challenging to tackle this issue in an observational setting because existing data are noisy and because it is hard to separate what proportion of the change in mental health is due to school closures rather than numerous changes that happened during the pandemic. This is especially difficult because school closures were often implemented in an entire country, resulting in a lack of spatial variation.

Even if there is spatial variation, the severity of the pandemic may influence mental-health outcomes rather than school closures. De Witte and François (2023) provided suggestive evidence that even in the absence of school closures—as was the case in Sweden during the COVID-19 pandemic—mental-health issues can arise despite limited learning deficits. Hence, although the literature attributes many negative consequences (e.g., the loss of learning opportunities, increased social isolation, reduced access to mental-health services, and increased risk of child abuse and neglect) to school closures, the causal interpretation of the results is questionable. Future research should therefore aim at disentangling the effect of school closures from the effect of the overall pandemic. Moreover, because the education-system characteristics may play a role, the limited empirical evidence often results in conflicting findings such that more research is needed.

## Policy Recommendations

The implementation of public-health policy during the pandemic has occurred with little if any acknowledgment of the psychological consequences of public-health measures on children. Therefore, we provide several recommendations for public-health policy based on earlier research that may help policymakers fully take children’s rights and needs into account during a public-health emergency.

First, an important point that became apparent during the review is that children from low socioeconomic backgrounds experienced the largest learning deficits. We recommend that policymakers take urgent action to address the educational inequalities exacerbated by the pandemic. One way to address this issue is to ensure that all children, particularly children from low socioeconomic backgrounds, have access to technology and the Internet to enable remote learning. The above-mentioned systematic review by De Witte and François (2023) found that countries that used information technology in education were more equipped to handle school closures during the COVID-19 pandemic. Policymakers should consider providing financial assistance to families to purchase technology equipment such as laptops and smartphones. To further support disadvantaged families, governments can provide funding for after-school programs or online tutoring services. Parents with a disadvantaged background may also require additional support, such as educational training and resources to assist their children’s learning at home (De Witte & François, 2023).

Second, the review has shown that school closures have exacerbated children’s mental health. We therefore recommend investing and implementing evidence-informed mental-health and resiliency programs in schools at a much larger scale than is the case currently. In the 2019–2020 school year, only 42% of K–12 schools in the United States offered mental-health treatment to students, such as psychotherapy or counseling (Schaeffer, 2022). Hamoda et al. (2021) showed that school mental-health programs can meet children’s mental-health needs resulting from school closures during the COVID-19 pandemic in a scalable manner once children return to school. The review also indicated that understanding the source of adverse effects on mental health is necessary for policy responses. Specifically, personality traits appear to matter in how individuals dealt with extreme shocks, and therefore, school mental-health programs should be personality tailored. For instance, more assistance could be provided to students with high neuroticism or low conscientiousness. Research also indicates that education may foster desirable personality traits (Obradović & Mazrekaj, 2023). Thus, the educational system should not only focus on improving cognitive skills but should also contribute to personality development. Such an approach will reduce mental-health problems that are due to extreme external situations in the future.

Third, we recommend fostering social and emotional learning (SEL) in schools. Research has shown that social-emotional competences can be taught and that schools are appropriate places to teach them (Weissberg, 2019). SEL programs improve students’ social-emotional skills and academic performance while reducing students’ conduct problems and emotional distress both for students with and without behavioral and emotional problems (Durlak et al., 2011; Payton et al., 2008; Taylor et al., 2017). Moreover, quality SEL programs yield an 11:1 return on dollars invested (Belfield et al., 2015). Even when implemented in distance learning because of school closures during the COVID-19 pandemic, SEL programs have been found to positively affect students’ social-emotional skills (Li et al., 2021). Johns Hopkins University (2022) keeps a database of evidence-based SEL programs that can be consulted by policymakers.

Finally, from a more moral rather than an evidence-informed perspective, people need to avoid the practice of labeling children who are growing up during the pandemic as “The COVID Generation,” “Generation C,” or “The Lost Generation.” According to the Pygmalion effect (Rosenthal & Jacobson, 1968), children may internalize their negative labels, leading to a self-fulfilling prophecy of low performance and psychosocial development. As stated by Rosenthal and Babad (1985), “When we expect certain behaviors of others, we are likely to act in ways that make the expected behavior more likely to occur” (p. 36). Indeed, Cox et al. (2018) showed that individuals were viewed more negatively when being labeled “Baby Boomer” than “older employee” in four workplace scenarios. This attention-grabbing negative framing should be replaced by positively framed statements. These children are not “lost,” they are only on a different, perhaps more challenging, path that creates opportunity to grow and should be treated as such. In line with this, persistently highlighting the struggles of parents with a marginalized background to deal with school closures may give the impression that these parents are not capable of looking after their children. It is of utmost importance to use positive framing to avoid destructive self-fulfilling prophecies.

In sum, given the large negative consequences that COVID-19 school closures have had on children’s learning and psychological outcomes, we suggest that schools should remain open except in the most extreme circumstances, closing as a very last resort. If such circumstances arise, more attention should be paid to students from marginalized groups who are most in need of intervention; evidence-informed, personality-tailored, mental-health and SEL programs should be implemented in schools; and generational labels should be avoided to ensure learning and psychosocial development of children. Psychological scientists should more explicitly communicate their findings and insights to warn policymakers about the devastating impact of extreme external shocks on the emotional well-being of individuals.

## Transparency

*Action Editor:* Dolores Albarracin

*Editor:* Interim Editorial Panel
